# Subscapularis- and deltoid-sparing vs traditional deltopectoral approach in reverse shoulder arthroplasty: a prospective case-control study

**DOI:** 10.1186/s13018-017-0617-9

**Published:** 2017-07-14

**Authors:** Alexandre Lädermann, Patrick Joel Denard, Jérome Tirefort, Philippe Collin, Alexandra Nowak, Adrien Jean-Pierre Schwitzguebel

**Affiliations:** 1 Division of Orthopaedics and Trauma Surgery, La Tour Hospital, Rue J.-D. Maillard 3, 1217 Meyrin, Switzerland; 2 Faculty of Medicine, University of Geneva, Rue Michel-Servet 1, 1211 Geneva 4, Switzerland; 3 Division of Orthopaedics and Trauma Surgery, Department of Surgery, Geneva University Hospitals, Rue Gabrielle-Perret-Gentil 4, CH-1211 Geneva 14, Switzerland; 4 Southern Oregon Orthopedics, Medford, Oregon USA; 5 Department of Orthopaedics and Rehabilitation, Oregon Health & Science University, Portland, Oregon USA; 6 Saint-Grégoire Private Hospital Center, Boulevard Boutière 6, 35768 Saint-Grégoire cedex, France

**Keywords:** Shoulder, Reverse shoulder arthroplasty, Length of stay, Deltopectoral approach, Subscapularis sparing, Approach, Cost-effectiveness, Results

## Abstract

**Background:**

With the growth of reverse shoulder arthroplasty (RSA), it is becoming increasingly necessary to establish the most cost-effective methods for the procedure. The surgical approach is one factor that may influence the cost and outcome of RSA. The purpose of this study was to compare the clinical results of a subscapularis- and deltoid-sparing (SSCS) approach to a traditional deltopectoral (TDP) approach for RSA. The hypothesis was that the SSCS approach would be associated with decreased length of stay (LOS), equal complication rate, and better short-term outcomes compared to the TDP approach.

**Methods:**

A prospective evaluation was performed on patients undergoing RSA over a 2-year period. A deltopectoral incision was used followed by either an SSCS approach or a traditional tenotomy of the subscapularis (TDP). LOS, adverse events, physical therapy utilization, and patient satisfaction were collected in the 12 months following RSA.

**Results:**

LOS was shorter with the SSCS approach compared to the TDP approach (from 8.2 ± 6.4 days to 15.2 ± 11.9 days; *P* = 0.04). At 3 months postoperative, the single assessment numeric evaluation score (80 ± 11% vs 70 ± 6%; *P* = 0.04) and active elevation (130 ± 22° vs 109 ± 24°; *P* = 0.01) were higher in the SSCS group. The SSCS approach resulted in a net cost savings of $5900 per patient. Postoperative physical therapy, pain levels, and patient satisfaction were comparable in both groups. No immediate intraoperative complications were noted.

**Conclusion:**

Using a SSCS approach is an option for patients requiring RSA. Overall LOS is minimized compared to a TDP approach with subscapularis tenotomy. The SSCS approach may provide substantial healthcare cost savings, without increasing complication rate or decreasing patient satisfaction.

## Background

The use of reverse shoulder arthroplasty (RSA) has increased substantially in recent years [1]. While the introduction of RSA has provided a solution for several previously untreatable conditions, as with most technological advancements, this has led to increased healthcare utilization and cost. Concurrently, from a macroscopic perspective, there has been growing interest within health systems to identify the most valuable or cost-effective treatments.

The bundled payment initiative has brought attention to examining multiple aspects of cost in the entire phase of care. In addition to implant cost, potential areas of cost savings include length of stay (LOS), complication and readmission rate, and postoperative rehabilitation center or physical therapy utilization. The impact of surgical approach for RSA on the aforementioned factors has not been previously studied. The most common surgical approach for RSA is a deltopectoral incision that includes a tenotomy or peel of the subscapularis to gain access to the glenohumeral joint. Recently, an approach which uses a deltopectoral incision but spares the subscapularis has been reported with good short-term clinical results [2]. Since this approach is subscapularis and deltoid sparing (SSCS), immediate active range of motion (ROM) without immobilization is allowed [2]. This fast-track rehabilitation protocol may therefore lead to cost savings.

The purpose of this study was to compare the clinical results of the SSCS and the TDP for RSA. The hypothesis was that the SSCS approach would be associated with decreased LOS, equal complication rate, and better short-term outcomes compared to a traditional deltopectoral (TDP) approach.

## Methods

### Patient selection

**Fig. 1:**
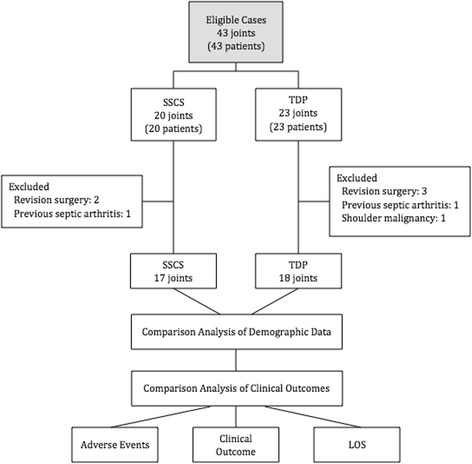
Patient selection

Between May 2013 and June 2015, all patients who had a primary RSA performed by one author (A.L.) with minimum follow-up of 3 months were considered potentially eligible for inclusion in this prospective case-control study that estimated the cost savings of a TDP approach compared to the SSCS approach. Patients with fractures, previous infection, shoulder malignancy, and revision surgery were excluded. Forty-three patients were considered potentially eligible for the study. Among them, five were excluded for revision shoulder arthroplasty, one for shoulder malignancy, and two for previous glenohumeral septic arthritis. Thus, there were 35 patients (35 RSAs) that met the study criteria. There were 18 patients in the TDP group and 17 patients in the SSCS group (Fig. 1). The study protocol was approved by the hospital ethics committee (AMG: 12–26), and all patients gave informed written consent.

### Surgical technique

All patients had general anesthesia with muscle relaxants used to facilitate glenoid exposure. Prior to skin incision, prophylactic intravenous antibiotics (cefazolin) were administered. In all cases, a deltopectoral incision was used [3]. The two approaches vary at the point of addressing the subscapularis tendon. If the subscapularis was torn, the remaining subscapularis and/or capsular tissue was tenotomized to gain access to the glenohumeral joint [4]. Conversely, if the subscapularis was intact, a SSCS approach was utilized as previously described [2]. For both approaches, the humeral head was cut with 20° of retroversion [5–7]. A circular baseplate (Aequalis Reversed; Tornier, Montbonnot, France) was implanted at the inferior edge of the glenoid. The glenosphere was usually eccentric to limit friction-type impingement in adduction, extension, and external rotation [8]. An onlay humeral stem with a final humeral inclination of 145° and an eccentric humeral plate was implanted. The eccentric infero-medial position was always used to limit arm lengthening and to maximize lateralization [9]. After closure of the incision, 160 mg of gentamicin mixed in 20 mL of saline was injected into the glenohumeral joint [10].

### Postoperative rehabilitation

In the case of a TDP approach, a standardized rehabilitation protocol was followed [11]. Patients were placed in a sling for 4 weeks. Passive ROM was initiated immediately, and active motion was allowed at 4 weeks. Strengthening was allowed at 8 weeks. With the SSCS approach, immediate active ROM was allowed with a sling for comfort only during the first few postoperative days [2] and strengthening was allowed at 6 weeks.

### Baseline characteristics and study variables

Baseline clinical characteristics extracted from the prospective database included age, sex, dominant hand, initial diagnosis (Hamada 1 to 2, Hamada 3 to 5, dislocation arthropathy, post-traumatic), previous shoulder surgeries, prior deltoid or subscapularis insufficiency, and baseline functional outcome and ROM. Baseline characteristics are summarized in Table 1.

**Table 1:** Baseline patient characteristics

	All prosthesis (*N* = 35)	SSCS approach (*N* = 17)	TDP approach (*N* = 18)	*P*
DRG insurance coverage	14	9	5	0.13
Failed cuff repair	3	0	3	0.08
Cuff tear arthropathy Hamada 1–2	14	10	4	0.03
Cuff tear arthropathy Hamada 3–5	10	5	5	0.91
Malunion	8	2	6	0.13
Age	78 ± 7	78 ± 7	78 ± 8	0.82
Sex (male)	8 (23%)	4 (24%)	4 (22%)	1
Dominant arm	18 (51%)	7 (41%)	11 (61%)	0.4
Previous surgeries	20 (61%)	5 (29%)	15 (94%)	0.23

*DRG* diagnosis-related group, *TDP* traditional deltopectoral, *SSCS* subscapularis and deltoid sparing

The primary outcome was LOS, including hospitalization and rehabilitation or post acute care. LOS during hospitalization was determined by the ability of the patient to return home. If unable, rehabilitation or post acute care was prescribed until the patient was able to independently return home. All costs were expressed in US dollars and estimated by adding the costs of the immediate postoperative hospital stay and rehabilitation stay. At our institution, the average cost of a hospital stay per night is approximately $1500 and the cost of a rehabilitation stay per night is approximately $647. Implant costs were excluded since we used the same implant in all cases and were not evaluating implant costs.

Secondary outcomes were adverse events (readmission and complication), number of postoperative physical therapy sessions, and clinical outcome at 3 months in terms of pain (visual analogue scale (VAS)), functional outcome (single assessment numeric evaluation (SANE)), and ROM in elevation, external rotation, and internal rotation) and at 12 months with Constant score [12]. Preoperative outcomes are summarized in Table 2.

**Table 2:** Preoperative outcomes

	All prosthesis (*N* = 35)	SSCS approach (*N* = 17)	TDP approach (*N* = 18)	*P*
Pain VAS	6.9 ± 2.3	6.9 ± 1.9	6.8 ± 2.7	0.84
SANE	32 ± 19	37 ± 14	27 ± 22	0.12
Forward elevation	95 ± 50	111 ± 58	75 ± 31	0.04
ER	19 ± 20	20 ± 21	19 ± 18	0.9
IR (median spinal height)	L4	L1	Sacrum	0.27

*ER* external rotation, *IR* internal rotation, *TDP* traditional deltopectoral, *SANE* single shoulder numeric assessment, *SSCS* subscapularis and deltoid sparing, *VAS* visual analogue scale

### Statistical analysis

Statistical analysis was performed with R v3.1.2 Portable (Free Software Foundation Inc, Vienna, Austria). Basic descriptive statistics (mean and percentages) were used for baseline clinical parameters and functional evaluation (VAS, SANE, and ROM). Clinical parameters of interest were compared between SSCS and TDS approach with two-tailed Student’s *t* or chi-squared test, when appropriate. Level of significance was set at *P* < 0.05.

## Results

There were no statistically significant differences between the two groups at baseline (Tables 1 and 2).

With the SSCS approach, the total length of stay was significantly shorter compared to the TDP approach. Hospitalization and rehabilitation stay costs were lower in the SSCS approach compared to the TDP approach (Table 3). There were no statistically significant differences between groups with respect to the number of physical therapy sessions. The SSCS approach was associated with a better functional outcome at 3 months in regard to SANE score and arm elevation. There was no statistically significant difference between the two groups in postoperative pain or range of internal and external rotation at 3 months postoperative (Table 4) and in Constant score at 1 year (68.1 ± 15.6 with SSCS approach vs 77.3 ± 12.9 with TDP approach; *P* = 0.07, respectively).

**Table 3:** Cost by surgical approach evaluated at 3 months post-surgery

	All prosthesis (*N* = 35)	SSCS approach (*N* = 17)	TDP approach (*N* = 18)	*P* value
Hospitalization stay	11.9 ± 10.2	8.2 ± 6.4	15.2 ± 11.9	0.04
Hospitalization costs (dollars)	13,600 ± 7900	10,500 ± 5200	16,400 ± 8700	0.02
Complication rate	1 (3%)	0 (0%)	1 (6%)	1
Number of outpatient care physical therapy sessions	14.1 ± 13.7	15.9 ± 17.9	12.4 ± 8.7	0.48

*TDP* traditional deltopectoral, *SSCS* subscapularis and deltoid sparing

**Table 4:** Clinical outcome evaluated at 3 months post-surgery

	All prosthesis (*N* = 35)	SSCS (*N* = 17)	TDP (*N* = 18)	*P* value
Pain VAS	1.2 ± 1.4	1.2 ± 1.5	1.2 ± 1.4	0.89
SANE	75 ± 15	80 ± 11	70 ± 16	0.04
Forward elevation	119 ± 25	130 ± 22	109 ± 24	0.01
ER	20 ± 24	25 ± 27	15 ± 21	0.29
IR (median spinal level)	L4	L1	L4	0.27

*ER* external rotation, *IR* internal rotation, *TDP* traditional deltopectoral, *SANE* Single shoulder numeric assessment, *SSCS* subscapularis and deltoid sparing, *VAS* visual analogue scale

During the follow-up period of 18 ± 11 months (range, 12 to 46 months), only one patient had a complication. This patient had a TDP approach and suffered a prosthetic dislocation 6 weeks postoperatively, which has been successfully managed with closed reduction. The same patient also experienced an acromial stress fracture that was managed conservatively. No subscapularis avulsion or iatrogenic tuberosity fracture was observed due to retraction during the SSCS approach.

## Discussion

The results of the current study support the hypothesis that the SSCS approach is associated with lower cost and equal complication compared to a TDP approach.

It is notable that the population is aging and older patients are the most likely to benefit from RSA [13]. However, the average hospital cost for shoulder arthroplasty is estimated to be $17,000 [14]. Consequently, it is important to find a solution to reduce the overall costs to provide continued access to RSA. LOS has recently been analyzed after shoulder arthroplasty in women, seniors, and comorbidity patients, with insurance coverage and diagnosis significantly contributing to increase in LOS [13, 15, 16]. In addition, hospital volume and surgeon experience have been associated with a lower LOS and cost compared to lower volume facilities and surgeons [17]. The current study examines an additional variable—that of surgical approach—which may affect cost. After controlling for preoperative and surgical variables, utilization of a SSCS approach compared to the current standard of a TDP approach for RSA resulted in an economic savings of $5881, corresponding to an average LOS of 7 days. By decreasing LOS and allowing earlier mobilization, such an approach may also help lower hospital-acquired infection rates [18], decrease risk factors for readmission [19], and improve patient satisfaction [20].

In addition to cost savings, the SSCS approach group was also significantly associated with a better functional outcome at 3 months compared to the TDP approach. At least four reasons could explain these differences. First, the subscapularis plays a crucial role in anterior elevation. Collin et al. previously demonstrated that the subscapularis is the most important rotator cuff muscle for elevation in native shoulders [21]. Although the RSA design partially changes the role of the subscapularis, an intact inferior subscapularis assures the joint protection necessary for ROM [22] and the superior subscapularis provides a positive vector force and function as an abductor [23]. Second, preservation of the subscapularis may improve internal rotation. A deficit in internal rotation is common after RSA, and while not well-studied, lack of healing of the subscapularis may partially account for this deficit. Third, if tenotomized or preoperatively torn, the subscapularis should be repaired whenever possible and protected in order to obtain healing as it plays a role in postoperative stability [24] at least in Medial Glenoid/Medial Humerus designs. Fourth, and finally, the SSCS approach allows immediate ROM. Immobilization has been shown to be associated with increased shoulder stiffness [25]. Postoperative immobilization following shoulder arthroplasty has been designed to balance the optimization of healing and prevention of stiffness. A 6-week period of immobilization is typically used to allow the tendon bone interface to progress through the normal healing phases of inflammation, proliferation, and remodeling [26]. After subscapularis repair in anatomic total shoulder arthroplasty, 4 weeks of immobilization lead to higher healing rates [27]. However, with a SSCS approach, immobilization may be avoided since there is no need to obtain subscapularis healing. Such early mobilization likely explains our superior clinical results in the SSCS group at short term. Nevertheless, the results were no different at 1 year.

Complications after RSA are related to etiology [28], prosthetic design [9, 29], arm lengthening [30, 31], and experience of the surgeon [32]. Traditionally, the rate of short-term complications after RSA is around 20% [28, 33, 34]. In this case-control series of 35 patients, the rate of short-term complications (3%) was lower than previously reported. In particular, we did not observe any technical problems with the SSCS approach. While further study with a larger cohort is needed, the early results with deltopectoral approach (with or without subscapularis sparing) are encouraging.

### Strengths and limitations

This prospective case-control study was the first to analyze the impact of a SSCS approach for RSA on cost. We observed substantial economic savings to the system, improved short-term results, and a minimal complication rate that may have the potential to change the standard for approach during RSA. However, there are several limitations that warrant discussion. First, different insurance coverages have been included in the study. The calculation was based on private division fees. Therefore, formal cost analysis was not possible for DRG patients [35] (i.e., patients without a private insurance coverage). Indeed, the cost of RSA for patients with DRG is not dependent of the length of the hospitalization stay. We consequently extrapolated the price regarding the loss of earnings for the hospital. Second, this study represents the learning curve and experience of one surgeon. Results could vary by learning curve and different geographical regions or health care systems. Concern has been expressed about cost savings from small changes in systems and techniques [36]. To date, no study has examined the economic effect of more widespread use of such approach, as it may not deliver significant savings at the macro scale. Effectively, it has not been proven that an anterosuperior approach [37], which involves the splitting of the deltoid muscle to avoid cutting the subscapularis tendon, is associated with lower cost or better functional results [38]. Third, we also recognize that SSCS approach might be challenging in certain cases (i.e., stiff shoulders) and may not be practical or possible in all circumstances. Fourth, our LOS was long. The latter is dependent of many factors, including patient factors (i.e., pain and ability to do ADLs) and health system factors. For example, in our country, our insurance system often imposes a minimum stay which artificially prolongs the LOS. In a recent study, Padegimas et al. demonstrated that LOS at orthopedic specialty hospitals is significantly shorter than at tertiary referral centers [39]. Their findings may be the result not only of fast-track rehabilitation and strict disposition protocols but also of less invasive surgical techniques. The cost-effectiveness of the SSCS approach is now even more apparent in our practice as patients are routinely discharging after only one to two nights in the hospital and no longer require an acute care stay and do not have therapy in the first 6 weeks postoperative. Fifth, due to the limited sample size, some of the comparisons performed might lack statistical power (type II error). Multicenter and prospective investigation will be necessary to determine the role of independent variables such as surgical approach, fast-track surgery, rehabilitation protocols, or health care systems.

## Conclusion

Using a SSCS approach is an option for patients requiring RSA. Overall, LOS is minimized compared to a TDP approach with subscapularis tenotomy. The SSCS approach may provide substantial healthcare cost savings, without increasing complication rate or decreasing patient satisfaction.

## Abbreviations

DRG: Diagnosis-related group
LOS: Length of stay
ROM: Range of motion
RSA: Reverse shoulder arthroplasty
SANE: Single assessment numeric evaluation
SSCS: Subscapularis and deltoid sparing
TDP: Traditional deltopectoral
VAS: Visual analogue scale
